# Parental coping with childhood cancer and its relationship with self‐construal: A survey in southeast Iran

**DOI:** 10.1002/hsr2.185

**Published:** 2020-08-19

**Authors:** Fereshteh Ghorbani, Sedigheh Iranmanesh, Farideh Razban, Mahlagha Dehghan

**Affiliations:** ^1^ Neonatal Intensive Care Nursing, Faculty of Nursing and Midwifery Kerman University of Medical Sciences Kerman Iran; ^2^ Department of Psychology Nursing, Faculty of Nursing and Midwifery Kerman University of Medical Sciences Kerman Iran; ^3^ Department of Critical Care Nursing, School of Nursing and Midwifery Kerman University of Medical Sciences Kerman Iran; ^4^ Department of Critical Care Nursing, Faculty of Nursing and Midwifery Kerman University of Medical Sciences Kerman Iran

**Keywords:** cancer, coping, Iran, parent, self‐construal

## Abstract

**Background:**

Childhood cancer is a major challenge for parapets. Parents are one of the main sources of emotional support for their child, but their ability to provide proper care during their child's illness and treatment depends entirely on the way they manage to cope with diagnosis and its outcomes. Parents' coping pattern seems to be affected by their perception of themselves or their surroundings.

**Aim:**

To investigate parents' coping strategies with childhood cancer and its relation with self‐construal.

**Methods:**

A total of 127 eligible parents participated in this descriptive correlational study.

**Results:**

Medical, social support, and family strategies were respectively helpful for parents. The interdependent self‐construal score was higher than the independent self‐construal score. A significant relationship was found between interdependent self‐construal and social support (*P* = .01).

**Discussion:**

It seems that individualists and collectivists' cultural context influence the usefulness of coping strategies. These differences should be considered in training of coping strategies.

## 1 INTRODUCTION

Cancer is one of the main causes of childhood mortality, with 300 000 new cases diagnosed among children aged 1 to 19 years worldwide.^1^ It has been estimated that 116 060 children diagnosed with cancer by 2019, and 1190 children died of cancer.^2^ About 4% of childhood mortality occurs under the age of 5 and 13% of mortality of children aged between 5 and 15 years are also related to cancer in Iran.^3^

One of the main sources of support for children with cancer is their parents, but it is dependent on how they manage to cope with diagnosis and outcomes.^4^ Coping is the use of cognitive and psychological resources, and behavioral strategies to eliminate, modify, and manage stressful conditions.^5^ Stressors and supporting sources do not just depend on the characteristics of the situation when caring for a child with cancer. People's different perceptions of stressors and strategies depend on the self‐perception and self‐construal.^6^ “Self” refers to how people perceive themselves, or what they know about themselves. In short, “self” is the answer to the question “who am I?” Self‐construal essentially affects the way people feel, perceive, behave, and attempt for a particular purpose.^7^ The individual's self‐construal varies from one culture to another.^8^

Hofstede divides the cultures into individualism and collectivism.^9^ It is assumed that individualists and collectivists have different self‐construal.^10^ In Western and individualistic cultures, the self‐construal is independent of others in the form of knowledge about difference, unity, and stability of the inner personality.^6^ Independent self‐construal is active when a person feels a particular identity and distinguishes himself from others,^7^ but the collectivistic concept of “self” includes others. Interdependent self‐construal is the extent to which people construct fundamental connections to others, focus on their relationships, and are concerned with the ways in which they can benefit their social group.^11^ Parents' inability to cope with stress can increase children's stress.^12^ Therefore, it is important to examine the factors affecting the parental coping with the child's illness such as self‐construal.

In a literature review, no study measured the relationship between self‐construal and coping with one's or a child's health problems. However, studies have been conducted recently in India,^8^ Turkey,^13^ Philippines,^14^ and the United States,^15^ which have different cultural and self‐construal conditions to determine the relationship between self‐construal and coping in general populations. In sum, the results of these studies have shown that the individuals' self‐construal, and whether they are individualistic or collectivistic, can influence the way an individual uses coping strategies in stressful conditions.^8^, ^13^, ^14^, ^15^ Therefore, this study aimed to determine the parental coping in caring for a cancer child and its relationship with their self‐construal in southeast Iran.

### 1.1 Context

Hofstede et al^16^ assessed the individualism index score for 76 countries, 0 was scored for the collectivistic countries and 100 for the individualistic countries. Iran, the 38th among 76 countries, was considered as a collectivistic country.^17^

Iranian Islamic culture emphasizes altruism and strong family relationships, which make Iranians more committed to their relatives, especially when a family member experiences an illness.^18^ Parents of cancer children face a variety of stressful sources, one of which is fear of death that is higher in men according to the terror management theory.^19^ The term cancer is equivalent to death in the Iranian culture.^20^, ^21^ Parents do not usually gain their information from healthcare team, which can be stressful for them.^22^ Unfortunately, there is currently no organized and principal structure for palliative and supportive care in Iran, with only five or six active centers.

## 2 METHODS

### 2.1 Study design and setting

The present study was descriptive correlational study. Parents of children with cancer referred to Afzalipour hospital and physicians' offices were studied in Kerman, the largest city in the southeast of Iran, from September 2017 to May 2018, Kerman provides oncology services to a large number of patients from other parts of the region.

### 2.2 Sample size and sampling

Parents of under 15‐year‐old children with cancer who were aware of the illness diagnosis with no psychological illness were participated in the study using convenience sampling method. The sample size was 127 parents based on the results of the pilot study on 30 parents, with a confidence coefficient of 95% and a power of 80%. In addition, the pilot study measured the relationship between the mean scores of the coping pattern and self‐construal (r = 0.24).

### 2.3 Measures

#### 2.3.1 Background information

A background information questionnaire consisted of parental characteristics such as age, occupation, level of education, living place, family relationship with the child, marital status, income, number of children, whether they had other child with cancer, whether they had lost any child, whether they had experienced the care for a cancer patient in their family, as well as the child's characteristics including age, gender, birth order, type of cancer, duration of illness, type of treatment, and frequency of referrals per month.

#### 2.3.2 Coping health inventory for parents

MacCubin et al designed the questionnaire in 1983. This questionnaire measures coping patterns among parents of chronically ill children. This questionnaire contains 45 items and three subscales. The subscales of this questionnaire are (a) maintaining family integration, cooperation, and an optimistic definition of the situation; (b) maintaining social support, self‐esteem, and psychological stability; and (c) understanding the healthcare situation through communication with other parents and consultation with the healthcare team. The answer to each question is based on a four‐point Likert scale ranging from 0 to 3 (“not helpful,” “minimally helpful,” “moderately helpful,” and “extremely helpful”), and the final score is between 0 and 135, which is obtained from the scores of all items. The higher the scores, the more effective the parental coping.^23^ The research team measured the validity and reliability of the questionnaire. The content validity index (CVI) was 90%. Reliability of the questionnaire was assessed by conducting a pilot study on 30 participants (also calculated in the final sample size), and Cronbach's alpha was .85. These results indicate the favorable validity and reliability of this questionnaire.

#### 2.3.3 Self‐construal scale

Singelis designed the self‐construal scale (SCS) in 1994 and revised it in 2000. The questionnaire consists of 30 items in the two areas of “independent self‐construal” and “interdependent self‐construal.” Each item is evaluated based on a seven‐point Likert scale, ranging from strongly disagree (1) to (7) strongly agree. Each participant will receive two points, one point for “independent self‐construal” and one point for “interdependent self‐construal.”^24^ This questionnaire has not been used in Iran, so the research team measured its validity and reliability. The CVI was calculated to be 90% for this questionnaire. Reliability of the questionnaire was assessed by conducting a pilot study on 30 participants (also calculated in the final sample size), and Cronbach's alpha was calculated .81. These results indicate the favorable validity and reliability of this questionnaire.

### 2.4 Data collection and analysis

The researcher referred to the study setting during different work shifts (morning, evening, and night) and started sampling after obtaining the permissions from the authorities of the oncology centers of Kerman. The questionnaires were completed in a self‐administered form and in case the parents were illiterate, they would have been completed by interviewing.

To analyze the data, we used SPSS v 21. Descriptive statistics were used to describe the characteristics of the samples (frequency, percentage, mean, and SD). Pearson correlation coefficient was used to calculate the correlation between coping strategies and self‐construal. Spearman correlation coefficient, independent *t* test, Mann‐Whitney *U*, ANOVA, and Kruskal‐Wallis tests were used to evaluate the mean scores of coping strategies and self‐construal in terms of background characteristics. Furthermore, all variables with *P* < .2 were simultaneously inserted into a full model of multivariate linear regression to assess the effect of different variables on the coping strategies or self‐construal. Significance level was set at .05.

### 2.5 Ethical considerations

The ethics committee of Kerman University of Medical Sciences approved the protocol of the study (No. IR.KMU.REC.1396.1897). The researchers explained research goals and protocol to the participants before their inclusion in the study, and if they had been willing to participate in the study, written informed consent would have been obtained from all eligible participants.

## 3 RESULTS

Table 1 shows the characteristics of the participants. The parents' mean total score of the coping health inventory was 86.3 ± 19.75. The “mean per item” was used to compare the domains because the number of items in different subscales of the questionnaire was not equal. The lowest mean score was related to “allowing myself to get angry” (0.59 ± 0.95) and the highest mean score was related to the item “believing in God” (2.98 ± 0.12) (Table 2). The interdependent self‐construal score (1.55 ± 0.24) was higher than the independent self‐construal score (1.29 ± 0.3). The total score of the SCS was obtained from the difference between the IND and INT scores (−0.26 ± 0.34). It should be noted that the higher the total score of the SCS, the higher the level of self‐construal, and vice versa, the lower the total score, the higher the interdependent self‐construal. Therefore, it can be concluded that the participants' interdependent self‐construal was greater than their independent self‐construal. A statistically significant relationship was found between self‐construal and coping health inventory for parents (CHIP) support (*P* = .01). From the perspective of those with more interdependent self‐construal, maintaining social support, self‐esteem, and psychological stability were more helpful, but no significant relationship was found among other coping strategies and the dimensions of self‐construal (Table 3).

**Table 1:** Participants' demographic characteristics

Variables	Mean (SD)
Parent's age (year)	36 (7.02)
Child's age (year)	6.7 (3.4)
Child's duration of cancer (month)	18.4 (20.7)
Visit times a month (No.)	1.8 (1.5)
Parent's gender	N (%)
Female	83 (65.4)
Male	44 (34.6)
Child's gender^a^	
Female	52 (40.9)
Male	74 (58.3)
Child's birth order^a^	
First	37 (43)
Second	26 (30.2)
Third	12 (14)
>Third	11 (12.8)
Child's type of cancer^a^	
Blood	71 (55.9)
Others	48 (37.8)
Child's type of treatment^a^	
Chemotherapy	89 (70.1)
Combination therapy	33 (26.0)
Marital status^a^	
Married	119 (93.7)
Divorce/widow(er)	5 (3.9)
Education level^a^	
Illiterate	8 (6.3)
Middle/high school	39 (30.7)
Diploma	45 (35.4)
Academic	22 (17.3)
Parent's job^a^	
Employed	46 (36.2)
Unemployed	78 (61.4)
Monthly family income^a^	
<1 million Toman	91 (71.7)
>1 million Toman	36 (28.3)
Number of children	
1	20 (15.7)
2	56 (44.1)
3	26 (20.5)
>3	25 (19.7)
City^a^	
Kerman	58 (47.5)
Other cites	64 (52.5)
Do you have another sick child?	
Yes	9 (7.1)
No	118 (92.9)
Have you ever lost a child?^a^	
Yes	13 (10.2)
No	113 (89.0)
Have you ever cared for a cancer patient in your family?^a^	
Yes	16 (12.6)
No	109 (85.8)

a In cases where the frequency is less than 127, missing value exists.

**Table 2:** Mean and SD of coping health inventory for parent scale

Subscale	Item description	Mean ± SD
*Family*: Maintaining family integration, cooperation, and an optimistic definition of the situation (13‐57) Row mean scores ± SD (37.5 ± 8.8)	1. Believing that my child(ren) will get better	2.08 ± 0.43
	3. Doing things with my children	2.32 ± 0.96
	6. Building a closer relationship with my spouse	2.54 ± 0.77
	8. Doing things with family relatives	1.69 ± 1.22
	11. Believing that my child is getting the best medical care possible	2.68 ± 0.62
	13. Doing things together as a family (involving all members of the family)	2.68 ± 0.69
	16. Engaging other members of the family with chores and tasks at home	1.98 ± 1.17
	18. Believing that the healthcare center/hospital has my family's best interest in mind	2.64 ± 0.60
	21. Being able to get away from the home care tasks and responsibilities for some relief	1.83 ± 1.05
	23. Eating	2.23 ± 0.94
	26. Purchasing gifts for myself and/or other family members	2.40 ± 0.76
	28. Working, outside employment	2.13 ± 0.98
	31. Talking to someone (not professional counselor/doctor) about my feelings	1.88 ± 1.04
	36. Building close relationships with people	1.91 ± 0.97
	38. Talking with other parents in the same situation and learning about their experiences	2.39 ± 0.85
	41. Reading more about the medical problem, which concerns me	2.31 ± 0.78
	43. Being sure that prescribed medical treatments for child(ren) are carried out daily at home	2.75 ± 0.52
	44. Talking with other individuals/parents in the same situation	2.47 ± 0.74
	45. Talking with the doctor about my concerns about my child(ren) with the medical condition	2.78 ± 0.44
Mean scores per item ± SD (2.3 ± 0.3)		
*Support*: Maintaining social support, self‐esteem, and psychological stability (18‐54) Row mean scores ± SD (35.2 ± 8.2)	2. Investing myself in my children	2.78 ± 0.45
	4. Believing that things will always work out	2.81 ± 0.50
	7. Talking with spouse about personal feelings and concerns	2.50 ± 0.87
	9. Believing in god	2.98 ± 0.12
	12. Trying to maintain family stability	2.76 ± 0.45
	14. Trusting my spouse (or former spouse) to support me and my child(ren)	2.82 ± 0.52
	17. Having my child with the medical condition seen at the clinic/hospital on a regular basis	2.84 ± 0.50
	19. Encouraging child(ren) with medical condition to be more independent	2.65 ± 0.64
	22. Getting away by myself	2.09 ± 1.01
	24. Sleeping	2.16 ± 0.98
	27. Concentrating on hobbies (art, music, jogging, etc.)	2.20 ± 0.88
	29. Becoming more self‐reliant and independent	2.45 ± 0.83
	32. Engaging in relationships and friendships which help me feel important and appreciated	2.18 ± 0.83
	33. Entertaining friends in our home	1.94 ± 0.94
	34. Investing time and energy in my job	1.95 ± 0.97
	37. Developing myself as a person	2.12 ± 0.84
	39. Talking with the medical staff (nurses, social worker, etc.) when we visit the medical center	2.61 ± 0.64
	42. Explaining our family situation to friends and neighbors so they will understand	1.72 ± 1.07
Mean scores per Item ± SD (2.4 ± 0.3)		
*Medical*: Understanding the medical situation through communication with other parents and consultation with healthcare team (3‐24) Row mean scores ± SD (13.5 ± 4.3)	5. Telling myself that I have many things I should be thankful for	2.94 ± 0.29
	10. Taking good care of all the medical equipment at home	2.44 ± 0.85
	15. Showing that I am strong	2.77 ± 0.54
	20. Involving in social activities (parties, etc.) with friends	1.94 ± 1.04
	25. Allowing myself to get angry	0.59 ± 0.95
	30. Keeping myself in shape and well‐groomed	2.46 ± 0.74
	35. Going out with my spouse on a regular basis	2.16 ± 0.83
	40. Reading about how other people with the same situation handle things	2.30 ± 0.90
Mean scores per Item ± SD (2.1 ± 0.4)		

**Table 3:** Correlations of self‐construal scale and CHIP scale

Variable	CHIP	Coping I	Coping II	Coping III
Self‐construal	r = 0.10	r = 0.07	r = 0.13	r = 0.04
	*P* = .27	*P* = .44	*P* = .16	*P* = .65
Independent self‐construal	r = 0.08	r = 0.03	r = 0.09	r = 0.15
	*P* = .32	*P* = .66	*P* = .31	*P* = .07
Interdependent self‐construal	r = 0.15	r = 0.10	R = 0.20	r = 0.07
	*P* = .09	*P* = .22	*P* = .01	*P* = .39

Abbreviations: CHIP, coping health inventory for parent; r, Pearson correlation coefficient.

The results of the univariate analysis showed that fathers (compared with mothers), married people (compared with the divorced and widows [er]), employed people (compared with the unemployed), parents with no other sick children, those who spent more time reading the Quran, parents who coped with their child's illnesses, and parents who considered their child's illness as a positive effect on their lives obtained higher coping strategy scores and considered coping strategies more helpful. No statistically significant relationship was found between other demographic variables and the score of coping strategies. Regarding the limited sample size and a large number of background variables, only the variables significant in the bivariate analysis were included in the multivariate regression model. Moreover, those with more than one million Toman income obtained higher scores of the coping strategies (by 9.43 scores) than those who earned below one million Toman. The score of coping strategies in people with no other chronically ill children was 16.35 points higher than those who had other children with cancer and these parents considered coping strategies more helpful (*P* < .001, *F* = 8.74, *F*, Adjusted *R* ^2^ = 0.29, *R* = 0.57).

A statistically significant relationship was observed between age and the mean score of self‐construal. However, no significant relationship was found between other demographic variables and self‐construal score. The variables in the bivariate analysis with a significant level of less than 0.2 were included in the multivariate linear regression model. Therefore, a significant relationship was found only between the number of children and self‐construal score. In other words, people with three children had lower self‐construal score than those who had one child (0.16), and they were more dependent (*P* = .33, *F* = 4.73, Adjusted *R* ^2^ = 0.03, *R* ^2^ = 0.2).

## 4 DISCUSSION

In the current study, a positive and significant correlation was found between social support, self‐esteem, psychological stability, and interdependent self‐construal, indicating that the higher the interdependent self‐construal, the more helpful the strategies. Searching in databases, we did not find a study with similar or opposite to this result. However, self‐construal theory has a profound effect on the recognition, emotions, and motivations of individuals in all cultures.^25^ Interdependent self‐construal emphasizes close relationships and membership in the group. Individuals with an interdependent self‐construal perceive “self” in connection with others.^26^ These people tend to rely on others.^27^ Dwivedi showed that individuals with more interdependent self‐construal are more vulnerable to interpersonal conflicts, and need to be supported by family and friends to deal with problems.^8^

The parents participated in the current study had a more interdependent self‐construal. So far, no study has been conducted to investigate self‐construal in Iran, but several studies have examined the difference of this variable in Asians, Europeans, and Americans, showing that Asians and, in general, the Orientals, have more interdependent self‐construal than Europeans, the Americans and, in general, the Western societies.^28^ Iran is a collectivistic country located in the southwestern part of Asia, so Iranians have more interdependent self‐construal.

The parents' mean total score of the coping health inventory was 86.3 (score range: 40‐135). The mean was higher than the results of previous studies in the United States.^29^, ^30^ In the present study, the highest mean score of coping strategies was related to the item “believing in God” (2.98 ± 0.12), which was consistent with the results of Nikfarid et al^31^ and Aliakbari‐Dehkordi et al.^32^ Studies consider religion as a defensive shield against stress.^33^ The majority of Iranians are Shiites. According to Qur'an, the Muslim holy book, believing in God has effects such as no sadness for difficulties and what one loses, which is achieved by trust in God and no dependency on material objects.^34^, ^35^

In the present study, the highest score of coping strategies was related to social support, self‐esteem, and psychological stability, which was consistent with the results of the studies by Nikfarid et al^31^ in Iran, and Hobdell et al^36^ in the United States. Social support can decrease an individual's psychological stress, and increase family ability to maintain integration, cooperation, and creation of a positive attitude.^29^ This dimension is helpful because Iranians have a close relationship with other people and especially their family members. Since 71.7% of the study population who were females and unemployed had income below 1 million toman, which is below the poverty line, much of the stress on these parents came from economic pressures, so such stress could reduce the ability of parental coping. Therefore, the support of parents by close relatives can be financially and emotionally effective in improving the parental coping.

It seems that parental access to social services and specialized support can reduce the stress associated with the diagnosis of a child's illness and enhance the parental coping.^37^ Meanwhile, the presence of supportive‐palliative care is very important. Mahak, a private organization in Iran, is the only charity fighting childhood cancer since 1991. However, the services of this institution alone do not meet the needs of all patients and their families due to the high population of children with cancer in Iran, the high cost of treatment, socio‐economic and social problems, and the chronicity of the disease.

High score of maintaining family integration, cooperation, and optimistic definition of the situation) in this study supports the results of some studies that examined the parental coping patterns in the care of children with chronic diseases such as cancer,^38^, ^39^, ^40^, ^41^ respiratory diseases,^42^ and metabolic disorders.^30^ These results suggest that coping patterns of the parents of children with cancer primarily preserve and strengthen the family structures,^40^ and the emotional bond of family members helps them achieve their strengths.^43^ In this study, married parents and people with no other chronically ill child considered coping strategies more helpful. This result can be due to the grater emotional bond and family support.

The low score of understanding the medical situation through communication with other parents and consultation with healthcare team in the present study is similar to the one obtained in the study of Nikfarid et al^31^ in Iran. They believe that low medical scores are due to the lack of proper healthcare systems to support and train families of the chronically ill children, lack of close relationship among patients, their families and the healthcare team, and lack of support from family members of the patients in Iran. The parents participated in the study of Aguilar‐Vafaie et al^38^ considered this dimension very effective, which is inconsistent with information obtained in the current study. Liu et al^44^ in China have shown that parents with higher education are more likely to consider this dimension helpful. The increasing education level of parents seems to be associated with an increasing sense of getting more information.^38^ The research team believes that the medical group does not have sufficient time and also is reluctant to provide information for the parents and does not encourage them to seek information.

Clinical nurses play a very important role in improving the parental coping, so they should be aware of coping strategies and self‐construal. Nurses must learn how to increase people's understanding of the meaning of “self” in different individuals. According to the results of this study, changes in management policies and managers' attention to the development of supportive‐palliative care in Iran could improve care for these children and their families. Moreover, the researchers have found no similar studies nationally or internationally. The publication of the results of this research can predispose to more questions and research at the national and international levels.

The present study had some limitations. The concern, fatigue, and low mood of the parents might have an impact on their response and motivation to participate in the study. To overcome this limitation, the researcher tried to deliver the questionnaire to parents when they had a proper mood and were psychologically ready to complete the questionnaire without any stress, so that more information could have been obtained accurately. Furthermore, a large number of questions might reduce the accuracy, so sampling was done in the best possible time, which was determined by participants. Finally, the results should be generalized to other populations with caution because the sampling in this study was only from the oncology centers of Kerman, Iran.

## 5 CONCLUSION

According to the results of this study, parental individualism or collectivism can be effective in using coping strategies, and “support” dimension of coping strategies is more helpful for parents with more interdependent self‐construal. Cultural dimensions are different from one country to another, and the results of studies conducted in the West cannot be used for the Orient. Cultural and familial differences should be considered in training of coping strategies. Educational sessions will be effective for peer groups in which parents with different religion, morality, education, family, age, and income share their experiences about how to cope with the child's illness. The results of the study showed that healthcare team, including doctors and nurses, should be trained about how to inform parents of childhood illness. Provision of information about the illness, treatment and its complications, and prevention methods are important in promoting the parental coping. Parents' awareness of the cancer and its cause will correct their views on cancer and eradicate the misconceptions about the disease as a result of social constraints. Correcting collective misconceptions about cancer can help parents accept illness and promote coping strategies. The establishment of a palliative care unit in the south of Iran will be effective in reducing the physical, mental, and financial problems of parents and in promoting coping strategies with child's illness.

## FUNDING 

None to be declared.

## ETHICS STATEMENT

The Kerman University of Medical Sciences Ethics Committee approved the study (IR.KMU.REC.1396.1897). The researcher gave explanations to the samples on their optional presence and that they can leave the study whenever they want. We also assured the participants that the collected information was confidential and would be used only for the research. Written informed consent was received from the parents. The study protocol was in accordance with ethical standards of Kerman University of Medical Sciences on human rights.

## CONFLICT OF INTEREST

The authors declare no conflicts of interest.

## AUTHOR CONTRIBUTIONS

Conceptualization: Fereshteh Ghorbani, Seddigeh Iranmanesh, Farideh Razban

Data curation: Fereshteh Ghorbani

Formal analysis: Mahlagha Dehghan

Methodology: Fereshteh Ghorbani, Seddigeh Iranmanesh, Farideh Razban, Mahlagha Dehghan

Software: Fereshteh Ghorbani

Supervision: Seddigeh Iranmanesh, Farideh Razban, Mahlagha Dehghan

Writing – original draft preparation: Fereshteh Ghorbani

Writing – review and editing: Fereshteh Ghorbani, Seddigeh Iranmanesh, Farideh Razban, Mahlagha Dehghan

All authors have read and approved the final version of the manuscript.

Mahlagha Dehghan had full access to all the data in this study and takes complete responsibility for the integrity of the data and the accuracy of the data analysis.

## TRANSPARENCY STATEMENT

The corresponding author, Mahlagha Dehghan, affirms that this manuscript is an honest, accurate, and transparent study, the important aspects of the study have not been omitted, and all discrepancies in the study have been explained.

## Data Availability

The data that support the results of this study are available from the corresponding author upon reasonable request.
